# An Open-Access Database for the Evaluation of Cardio-Mechanical Signals From Patients With Valvular Heart Diseases

**DOI:** 10.3389/fphys.2021.750221

**Published:** 2021-09-30

**Authors:** Chenxi Yang, Foli Fan, Nicole Aranoff, Philip Green, Yuwen Li, Chengyu Liu, Negar Tavassolian

**Affiliations:** ^1^School of Instrument Science and Engineering, Southeast University, Nanjing, China; ^2^Mount Sinai Morningside Hospital, New York, NY, United States; ^3^Interventional Cardiology and Vascular Medicine, Sorin MedicalMount Sinai Morningside Hospital, New York, NY, United States; ^4^Department of Electrical and Computer Engineering, Stevens Institute of Technology, Hoboken, NJ, United States

**Keywords:** database, seismo-cardiogram, gyro-cardiogram, electrocardiogram, valvular heart disease

## Abstract

This paper describes an open-access database for seismo-cardiogram (SCG) and gyro-cardiogram (GCG) signals. The archive comprises SCG and GCG recordings sourced from and processed at multiple sites worldwide, including Columbia University Medical Center and Stevens Institute of Technology in the United States, as well as Southeast University, Nanjing Medical University, and the first affiliated hospital of Nanjing Medical University in China. It includes electrocardiogram (ECG), SCG, and GCG recordings collected from 100 patients with various conditions of valvular heart diseases such as aortic and mitral stenosis. The recordings were collected from clinical environments with the same types of wearable sensor patch. Besides the raw recordings of ECG, SCG, and GCG signals, a set of hand-corrected fiducial point annotations is provided by manually checking the results of the annotated algorithm. The database also includes relevant echocardiogram parameters associated with each subject such as ejection fraction, valve area, and mean gradient pressure.

## Introduction

Valvular heart diseases (VHDs) are one of the most common cardiovascular diseases around the globe, accounting for 20% of cardiac surgical procedures performed in the United States (Benjamin et al., 2018). In China, the weighted prevalence of VHD is 3.8%, with an estimated 25 million patients (Yang et al., 2021). The continuous evaluation of cardiovascular activities during daily life has become an essential part of the current healthcare system to diagnose cardiovascular diseases (Barrett et al., 2014). For instance, wearable devices that record electrocardiograms (ECG), such as the conventional Holter monitor and newly-developed single-lead ECG patches are practiced for daily monitoring of cardiovascular activities to detect diseases such as arrhythmias and atrial fibrillation (Cai et al., 2020). These devices generally record the ECG signals from 24h to 14days while the subject is outside the clinic and is free to move. However, ECG-based devices are not effective in detecting VHDs. Current methods for diagnosing VHDs include clinical evaluations such as echocardiography, magnetic resonance imaging, and computed tomography scan (Faggiano et al., 2012). These methods are constraining and not suitable for out-of-the clinic monitoring. Currently, there is no device that can play a role similar to that of the Holter monitor in arrhythmia detection for VHD management.

Cardio-mechanical sensing is a relatively new sensing modality that can non-invasively monitor cardiovascular functions in a wearable form factor. It represents the chest vibrations induced by the heartbeat in the form of linear acceleration and rotational speed, which are named as seismo-cardiogram (SCG) and gyro-cardiogram (GCG) signals, respectively (Inan et al., 2014; Tadi et al., 2017). These signals can be detected by placing an inertial measurement unit (IMU), consisting of an accelerometer and a gyroscope, on the chest wall of subjects. The acquisition and analysis of cardio-mechanical signals, also known as mechanocardiograms (Kaisti et al., 2018a), have been widely studied and are reported to have potential value in clinical and home applications. These modalities have been used for the classification of several types of cardiovascular diseases including aortic stenosis (AS; Yang et al., 2020a), coronary artery disease (Korzeniowska-Kubacka et al., 2005), myocardial infarction (Iftikhar et al., 2018), atrial fibrillation (Hurnanen et al., 2016), and heart failure (Inan et al., 2018). However, the development and comparative analyses of algorithms have been hindered by the lack of high-quality, validated, and standardized open databases of cardio-mechanical signals. The review in Inan et al. (2014) also suggests that the availability of databases is one of the major problems in SCG-related research.

Prior to the submission of this manuscript (July, 2021), there were only two open-access databases for cardio-mechanical modalities. The first database is the Combined measurement of ECG, Breathing and Seismocardiograms (CEBS) available from PhysioNet (Goldberger et al., 2000; García-González et al., 2013; García-González and Argelagós-Palau, 2013). ECG, respiration, and SCG were recorded using a wired data acquisition system in a supine position from 20 presumed healthy volunteers, reporting an average age of 24.7years. The measurement of each subject consists of three phases, including a basal phase of 5minutes, a second phase of 50minutes with music playing at rest, and the last phase of 5minutes at rest. This database includes only one axis of SCG, and therefore is not suitable for studies/algorithms that utilize multi-axes SCG and GCG signals. Another database is the mechanocardiograms with ECG reference (MCGER), available from the IEEE DataPort (Kaisti et al., 2018b). This dataset is associated with the study of multinational cardio-mechanical signals reported in IEEE Sensors Journal (Kaisti et al., 2018a). It consists of 29 recordings of 3-axis SCG and GCG with ECG reference. Similar to the CEBS, the cohort of this dataset are young, healthy subjects that are 29years old on average.

There are several problems to be addressed with the currently available databases. The first is that they only include healthy subjects and are therefore not useful for evaluating classification algorithms of cardiovascular diseases. Secondly, the two cohorts are young. Age-related factors might bias the results from these databases. Moreover, the main population that is at high risk of cardiovascular diseases are senior citizens. Lacking the data from this target demographic group may have a negative impact on the development of data-driven models. Lastly, the current databases’ sizes are relatively small compared to those for ECG research (Greenwald, 1990; Clifford et al., 2017; Cai et al., 2020). This problem may lead to the overfitting of machine-learning algorithms, prohibiting the development of robust models with sufficient generalization capability.

This article presents a database of cardio-mechanical signals from patients with VHDs in the United States and China. In addition to the six channels of 3-axis SCG and 3-axis GCG recordings, detailed information about the patients including demographics (number, age, and gender), meta information of the recordings, axes definitions, synchronously-recorded ECG signals, and echocardiogram reports are also included in the database. A set of hand-corrected annotations is also provided by manually checking the recordings annotated by a computer. The database can be accessed by visiting the website described in the Data Availability Statement section. To the best of our knowledge, this is the first database of SCG and GCG signals acquired from cardiovascular patients. It is also obtained from a more comprehensive age range than the current databases, especially as it contains the under-represented senior citizen group. This database could be a promising additional component to the currently available databases to fill the gaps and assemble a more extensive database, promoting the study of SCG and GCG signals, as well as the study on the relationship between SCG, GCG signals, and the synchronously-recorded ECG signals.

This manuscript is organized as follows. Section Materials and Methods introduces the methods of data collection and annotation. The description of data is also included in this section. Several suggestions on the interpretation of the database are presented in Section Suggestions for the Interpretation of the Database.

## Materials and Methods

### Assembly of the International Database

The presented database includes international sources from two clinical sites, the Columbia University Medical Center (CUMC) located in New York City, NY, United States, and the First Affiliated Hospital of Nanjing Medical University (FAHNMU), Nanjing, Jiangsu Province, China. Data were collected between the years 2017 and 2021. Some of the recordings have been evaluated in previous literature although not open to the public at the time. Details of the data collection procedures at the two sites are mentioned below.

#### Data From Patients in the United States

There were two cohorts at CUMC. The first cohort included 21 patients who were diagnosed with aortic stenosis (AS) and other co-existing VHDs. Data were collected in 2018, and the study of this cohort was presented in Yang et al. (2019, 2020a). The second cohort consisted of nine AS patients who needed transcatheter aortic valve replacements. Data collection was completed in 2019, and the presentation of the corresponding study was in Yang et al. (2020b). All the data were collected either in the cardiac care unit or the pre-operative room of the CUMC, in collaboration with Dr. Philip Green.

In summary, 30 patients with AS and co-existing VHDs were included in the United States dataset. It is also worth mentioning that all the recordings were collected before any treatment. The dataset was processed and analyzed at Stevens Institute of Technology, Hoboken, NJ, United States. The patients of this dataset are named as UP-XX, in which XX ranges from 01 to 30 and represents the deidentified ID of the patient.

#### Data From Patients in China

The cohort at FAHNMU included 70 patients who were diagnosed with VHDs. The data collection was conducted in the Department of Cardiovascular Surgery from 2020 to 2021, in collaboration with Dr. Le Geng. All the recordings were measured before any treatment, and the data were processed and annotated at Southeast University, Nanjing, Jiangsu Province, China. The patients in this dataset are named as CP-XX, ranging from 01 to 70.

#### Summary of the Database

The database consists of 100 patients in total from the United States and China. The age of the subjects ranges from 29 to 97years old, with an average of 68years and a standard deviation of 14years. The subjects’ heights vary from 139.7 to 186.0cm, with 165cm on average and 9cm on standard deviation. The weights range from 44 to 118kg, showing an average of 69kg and a standard deviation of 13kg. The summary and comparison of demographic information to state-of-the-art databases are shown in Table 1. It is shown that the presented database is larger than the available databases in size and covers a wider variety of ages, weights, and heights. Our database contains more entries and covers a wider variety of ages, weights, and heights. Ours is also more balanced between the genders, with 41% female and 59% male.

**Table 1:** Summary and comparison of the demographic information of databases.

Database	Cardio-mechanical modalities	Additional modalities	Cohort size (female: male)	Subject conditions	Age (years), range (average±standard deviation)	Weight (kg), range (average±standard deviation)	Height (cm), range (average±standard deviation)
Combined measurement of ECG, breathing and seismocardiogram (CEBS) database	SCG	Breathing and ECG	17 (6:11)	Healthy	Unknown (24.7±3.9)	N/A	N/A
Mechanocardiograms with ECG references	SCG and GCG	ECG	29 (0:29)	Healthy	23–41 (29±5)	60–98 (76±11)	170–190 (179±5)
Presented database	SCG and GCG	ECG	100 (41:59)	VHD patients	29–97 (68±14)	44–118 (69±13)	140–186 (164±9)

Along with the demographic information, VHD labels were also provided. The subjects were marked with five VHD labels by cardiologists, including the existence of moderate or greater AS, aortic valve regurgitation (AR), mitral valve stenosis (MS), mitral valve regurgitation (MR), and tricuspid valve regurgitation (TR). The existence of each VHD is marked with 1 and its non-existence with 0. Among all subjects in the database, 39 have moderate or greater AS, 28 are diagnosed with AR, 23 have MS, 51 reported with MR, and 40 have TR. Most of the subjects are labeled with more than one VHD, covering a large variety of mixed VHD conditions. Moreover, parameters from echocardiogram reports are also included as metadata for each subject. The parameters include the dimensional parameters and valve area, mean gradient, and peak velocity, etc. More details could be found in the summary file of the database.

### Experimental Protocol

An off-the-shelf sensor node (Shimmer 3 ECG module, Shimmer Sensing, United Kingdom) was used as the recording device during all measurements. The device comprises 3-dimensional micro-electromechanical system (MEMS) accelerometer and gyroscope that detect and record SCG and GCG signals, respectively. The device also includes a bio-potential circuit that simultaneously records the ECG signal. The device is set to SD-writing mode, in which the data are first recorded to the local SD card and imported to a computer after the experiment. The range of the gyroscope is set to ±250 degrees per second, and the gain of the ECG amplifier is set to 4. The recordings from CP-01 to CP-70 and UP-01 to UP-21 are measured with a sampling rate of 256Hz, and the recordings from UP-22 to UP-30 are measured with a sampling rate of 512Hz.

**Figure 1:**
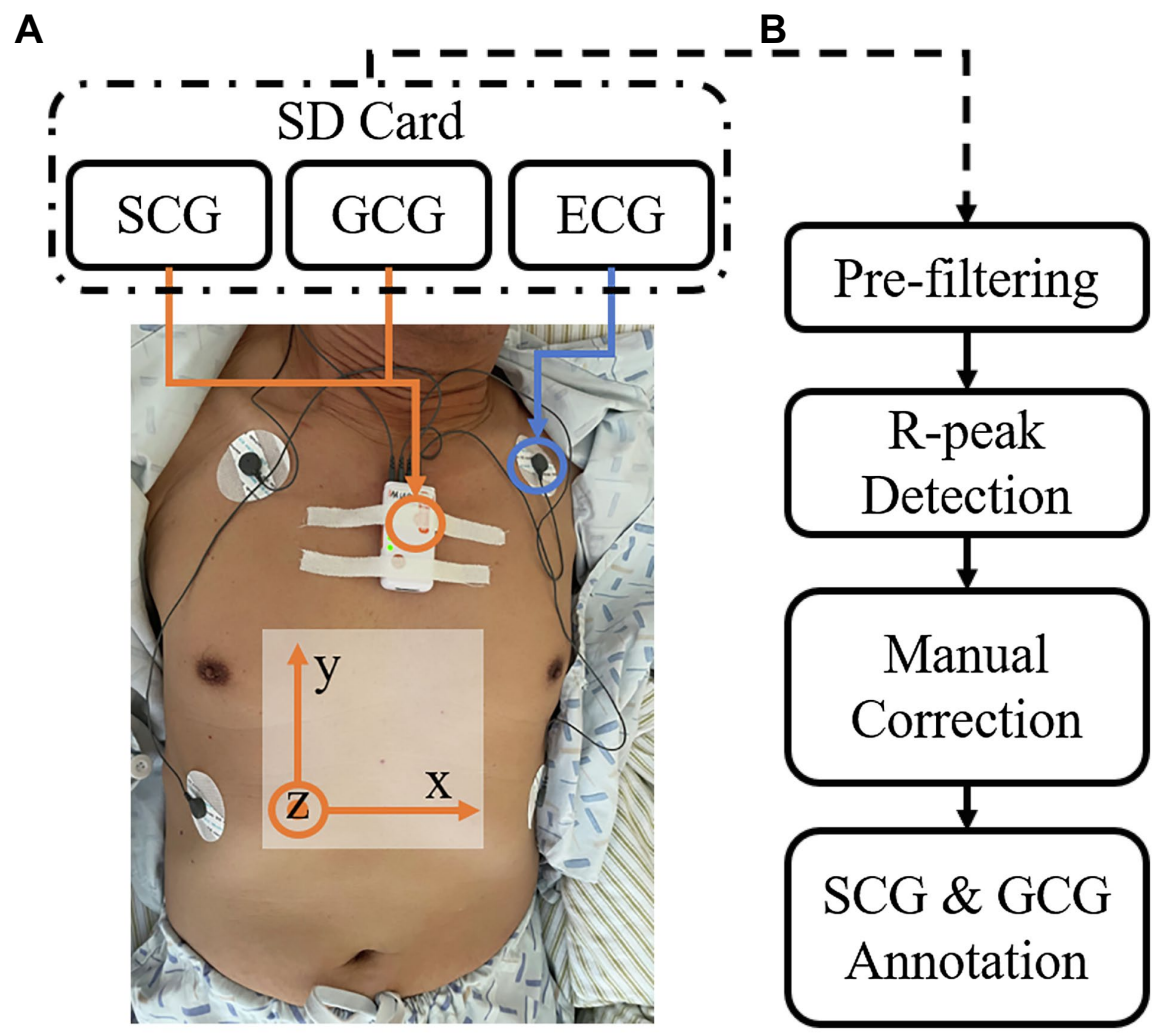
**(A)** Representative measurement setup and the axes definitions. **(B)** The annotation process of the ECG, seismo-cardiogram (SCG), and gyro-cardiogram (GCG) recordings.

Figure 1A demonstrates the setup and axes definitions of the measurements from a representative subject. The device is attached to the sternum using medical tapes or chest straps. Four medical electrodes are attached to the skin of the subject in a limb-ECG setup and connected to the device using lead wires. Nine biophysical signal channels are collected from ECG, SCG, and GCG modalities. The LA-RA, LL-LA, and LL-RA standard ECG channels are recorded *via* the limb-ECG setup. As shown in Figure 1A, three axes of SCG and GCG are extracted based on the same axis system, in which the *x-axis* represents the shoulder-to-shoulder direction, the *y-axis* refers to the head-to-foot direction, and the *z-axis* shows the dorso-ventral direction of the body.

During measurements, subjects are asked to stay in a supine, awake position. They are also guided to perform normal respiration without holding their breaths. The effective measurement from each subject lasts longer than 4min, reporting 6min 48s on average and 11h 19min 21s in total.

The patient experimental protocol was approved by the Institutional Review Board of CUMC under protocol number AAAR4104 as well as the Institutional Review Board of FAHNMU under the approval number 2019(878). A consent form was signed by each subject before measurements. All the metadata were deidentified before making it publicly available.

### Data Description and Annotation

#### Database File Description

There are eight files for each subject in our database. They are the raw recordings from the device in comma-separated value format (CSV), the extracted column vectors in MATLAB Data format (MAT), ECG annotations in MAT and JavaScript Object Notation formats (JSON), SCG annotations in MAT and JSON, and GCG annotations in MAT and JSON, respectively. The file names indicate the corresponding subject number and file content. For instant, the files for subject UP-01 are “UP-01-Raw.csv,” “UP-01-Vectors.mat,” “UP-01-ECG.mat,” “UP-01-SCG.mat,” “UP-1-GCG.mat,” “UP-01-ECG.json,” “UP-01-SCG.json,” and “UP-1-GCG.json.” More information could be found in the “Read_ME.txt” file of the database.

#### Data Annotation

A semi-automatic process annotated all nine channels of biophysical data as demonstrated in Figure 1B. Firstly, the signals are pre-filtered to remove baseline wandering and unwanted frequency bands. The ECG signals are band-pass filtered from 0.8 to 30Hz, and the SCG and GCG signals are band-pass filtered from 0.8 to 25Hz. After the pre-filtering, an analysis segment from the raw recordings was manually selected by trimming the start and end of the recordings. This step removes the signals that were recorded during sensor attachment and removal. The start time and duration for each subject are written in the summary file of the database.

The signal segments are then annotated to mark the representative heartbeat markers from each modality by using a modified version of the R-DECO toolbox (Moeyersons et al., 2019). Firstly, the R-peaks of ECG are marked by using a modified Pan-Tompkins algorithm. The peaks are then manually checked to ensure a reliable R-peak reference. Based on the timestamp of the detected R-peaks, an algorithm that searches the nearby maxima peaks was applied to SCG and GCG waveforms. More details of this searching algorithm can be found in the literature (Yang and Tavassolian, 2017). These peaks represent the aortic opening of the heart. It is to be noted that the SCG and GCG peaks were not manually checked. All ECG, SCG, and GCG peaks were stored by the timestamps of the peak location, in the format of *hh:mm:ss.ssss* (hour-minute-second). The vector that contains the peak information is stored in the corresponding MAT file as introduced in the previous subsection.

## Suggestions for the Interpretation of the Database

### Interpretation of the Annotations

The R-peak annotations of ECG were provided with a high confidence level by conducting manual checking twice. These peaks are presented as reliable heartbeat information for the users to facilitate the segmentation of the recordings and the development of ECG-based annotation algorithms. On the other hand, the corresponding SCG and GCG peaks were not manually checked due to two main reasons. The first is that the definitions of multi-axes SCG and GCG peaks are not standardized and remain an open question. Therefore, it is not suitable to manually check the peaks based on only the authors’ understanding and claim them as “ground truth.” Secondly, the waveforms from VHD patients reveal various morphological differences compared to those from healthy subjects. The current peak detection results show the representative performance of a simple peak-search algorithm to these abnormal signals and could be used as references for algorithm development. The users of this database are encouraged to apply their customized algorithm for peak detection.

### Interpretation of the Meta Information and Labels

There are mainly two groups of meta information. The first group is the VHD labels, and the second is the echocardiogram parameters. The VHD labels were given by cardiologists based on the overall symptoms of the subjects, including their echo reports. The users are encouraged to conduct multi-label or multi-class analyses based on the provided VHD labels. On the other hand, the echocardiogram parameters only represent the conditions of the heart at the time of the echocardiogram measurement, and the biophysical recordings are NOT recorded simultaneously. Although the time of echocardiogram is close to the measurement time of the biophysical signals, they are not intended for beat-to-beat analyses.

## Data Availability Statement

The datasets presented in this study can be found in online repositories. The names of the repository/repositories and accession number(s) can be found at: https://doi.org/10.5281/zenodo.5279448.

## Ethics Statement

The studies involving human participants were reviewed and approved by the IRB Review Board of CUMC and SEU. The patients/participants provided their written informed consent to participate in this study.

## Author Contributions

CY contributed to the original idea, planning and organization of the experiments, data collection, data annotation, and manuscript writing. FF contributed to data interpretation and analysis. NA and PG contributed to the data collection of the United States cohort. CL and NT contributed to the original idea, planning and organization of the study, and manuscript writing. All authors contributed to the article and approved the submitted version.

## Funding

The work in China is supported by the Distinguished Young Scholars of Jiangsu Province (BK20190014), the National Natural Science Foundation of China (62101120, 62001111, and 81871444), and the Primary Research & Development Plan of Jiangsu Province (BK20210208, BK20200364, and BE2017735). The data collection and processing in the United States are funded by the National Science Foundation (NSF) under award number 1855394.

## Conflict of Interest

The authors declare that the research was conducted in the absence of any commercial or financial relationships that could be construed as a potential conflict of interest.

## Publisher’s Note

All claims expressed in this article are solely those of the authors and do not necessarily represent those of their affiliated organizations, or those of the publisher, the editors and the reviewers. Any product that may be evaluated in this article, or claim that may be made by its manufacturer, is not guaranteed or endorsed by the publisher.
